# Extracellular and Intracellular Regulation of Calcium Homeostasis

**DOI:** 10.1100/tsw.2001.489

**Published:** 2001-12-22

**Authors:** Felix Bronner

**Affiliations:** Department of BioStructure and Function, University of Connecticut Health Center, Farmington, CT 06030-6125, USA

**Keywords:** vitamin D, duodenum, jejunum, ileum, skeleton, osteoblasts, osteoclasts, Ca-sensing receptor, calcium entry structure

## Abstract

An organism with an internal skeleton must accumulate calcium while maintaining body fluids at a well-regulated, constant calcium concentration. Neither calcium absorption nor excretion plays a significant regulatory role. Instead, isoionic calcium uptake and release by bone surfaces causes plasma calcium to be well regulated. Very rapid shape changes of osteoblasts and osteoclasts, in response to hormonal signals, modulate the available bone surfaces so that plasma calcium can increase when more low-affinity bone calcium binding sites are made available and can decrease when more high-affinity binding sites are exposed. The intracellular free calcium concentration of body cells is also regulated, but because cells are bathed by fluids with vastly higher calcium concentration, their major regulatory mechanism is severe entry restriction. All cells have a calcium-sensing receptor that modulates cell function via its response to extracellular calcium. In duodenal cells, the apical calcium entry structure functions as both transporter and a vitamin D–responsive channel. The channel upregulates calcium entry, with intracellular transport mediated by the mobile, vitamin D–dependent buffer, calbindin D_9K_, which binds and transports more than 90% of the transcellular calcium flux. Fixed intracellular calcium binding sites can, like the body's skeleton, take up and release calcium that has entered the cell, but the principal regulatory tool of the cell is restricted entry.

## INTRODUCTION

Calcium is the fifth most abundant element on earth[1]. Its location and concentration in the mammalian body are closely regulated. The blood plasma concentration of calcium is 2.5 mM[1], with a normal variation of no more than 20%. Approximately half of the plasma calcium is bound, largely to blood proteins, with albumin being the major ligand. It is the extracellular ionic calcium concentration that is closely regulated at around 1.2 mM. The intracellular free calcium concentration is typically between 50 and 100 nM; that is, it is about $10^4$ times lower than the ionic calcium concentration outside the cell membrane. Clearly, then, the cell must have mechanisms to keep calcium out.

Terrestrial animals must accumulate calcium to maintain their internal skeleton. Calcium thus represents a fascinating and unusual situation: its concentration is regulated at two vastly different levels, while at the same time it is accumulated in the skeleton. To reach the skeleton, calcium that has been ingested must pass through and between the intestinal cells. When absorbed calcium reaches the extracellular fluids bathing the cells, it will, at least transiently, raise their calcium concentration. It will do so also in the cell, but not proportionately. In this article I shall discuss the mechanisms by which plasma calcium and intracellular calcium are regulated.

## CALCIUM-REGULATING MECHANISMS

Calcium enters the body via foods and, following digestion, is absorbed from the intestine by two routes: a transcellular route dominant in the duodenum, and a paracellular route that assumes functional significance in the lower half of the small intestine[2]. The transcellular route is upregulated when calcium intake is low and downregulated when the food calcium supply is abundant[1,2,3,4]. The paracellular route depends on tight junction permeability and on the difference between the calcium concentration in the intestinal lumen and that in the body fluids. But because the chyme remains in the jejunum, and especially in the ileum, for relatively long periods — on the order of 4 h — even small differences in concentration allow reasonable amounts of calcium to be absorbed. The transcellular route, on the other hand, assumes functional significance only when calcium intake is low. Under these conditions, transcellular calcium transport is upregulated and a substantial fraction of the limited amount of calcium that enters the intestine is absorbed in the duodenum, with body uptake increased.

When food calcium is abundant, downregulation of the transcellular route has but a minor effect on total calcium absorption, as most calcium is absorbed in the lower intestine via the paracellular route. Inasmuch as only transcellular calcium transport is vitamin D–dependent, calcium entry into the body via food intake is subject to very modest regulation, which in turn is due only to 1,25-dihydroxyvitamin $D_3$, the most active vitamin D metabolite.

The inference that vitamin D–dependent transcellular calcium transport plays only a modest role in regulating total calcium entry into the organism is borne out by studies[5] demonstrating that when fed a high-calcium diet (2% Ca, 1.25% P, 20% lactose) vitamin D–receptor knockout mice did not differ in serum calcium, femoral calcium, and serum parathyroid hormone levels from wild-type mice in control groups, although the serum 1,25-dihydroxyvitamin $D_3$ levels of the knockout mice were less than half those of the controls. The calcium parameters of the knockout mice were, however, significantly lower than those of the controls when the groups were fed low or intermediate calcium diets (without lactose).

It is now widely recognized that transcellular calcium transport is a three-step process, consisting of calcium entry at the brush border, facilitated diffusion through the cell interior, and active extrusion at the basolateral membrane[2,6]. Calcium extrusion is mediated by the Ca-ATPase[6] and, to a minor degree, by a $Na^+/Ca^{++}$ exchanger[2,6]. It has been shown experimentally[2] that the extrusion capacity is more than adequate; therefore, this step is not rate limiting in the overall transport, although it is upregulated by vitamin D[7]. Intracellular self-diffusion of free calcium ions is too slow to account for the experimental rate of transcellular calcium movement[2]. Instead, calbindin $D_{9K}$, a cytosolic protein discovered by Wasserman and colleagues[8], has been shown to act like an intracellular ferry, increasing the rate of calcium diffusion by binding calcium[2,9]. Indeed, the rate of maximum calcium flux through the duodenal cell is a linear function of the cellular content of calbindin $D_{9K}$[2].

**TABLE 1**
**Effects of Vitamin D on Transcellular Calcium Transport in Intestine**

| Step | Mechanism or Structure | Effects of Vitamin D | Mechanism |
|---|---|---|---|
| Entry across brush border | Down electrochemical gradient of $Ca^{2+}$ via transporter and channel | Enhances by up to 90% | Mediated by calcium channel |
| Binding to fixed cellular sites | Golgi apparatus; rough endoplasmic reticulum; mitochondria | Enhances by 100% | Unknown |
| Intracellular movement | Self-diffusion and diffusion on a mobile buffer | Facilitates diffusion up to 70-fold | Biosynthesis of soluble CaBP, which acts as a Ca ferry |
| Extrusion | Pumping against a gradient | Increases action 2- to 3-fold | Perhaps by increased synthesis of Ca ATPase |

*Note:* CaBP : calbindin $D_{9k}$; adapted from Bronner et al.[2]

In facilitating intracellular calcium diffusion, calbindin $D_{9K}$ binds more than 90% of the transcellular calcium that enters the cell and thus also acts as a mobile buffer that keeps the intracellular calcium concentration low, notwithstanding a large steady-state calcium flux through the cytosol.

Cellular calcium entry, when evaluated by means of isolated brush border membrane vesicles[10], increases in proportion to the difference in luminal calcium concentration. Because of the vast gradient between luminal and intracellular calcium concentrations, it is obvious that calcium entry into the cell must be severely restricted. Recently an apical calcium entry mechanism has been identified, termed ECaC in rabbit duodenum[11,12], and CaT1 in rat duodenum[13]. The two proteins are either very similar or identical, with a substantial but not total vitamin D dependence[5,11,12,13].

The major regulator of transcellular calcium transport is vitamin D[2] which, after biotransformation to 1,25-dihydroxyvitamin D, acts to augment all three processes—entry, diffusion, and extrusion—albeit not to the same extent (Table 1). The biosynthesis of 1,25-dihydroxyvitamin D is upregulated under conditions of low or deficient calcium intake and downregulated when calcium intake is abundant.

The reason downregulation of the active, transcellular calcium transport process has relatively little effect on the overall amount of calcium that is absorbed under conditions of calcium abundance is twofold, as implied above: transit or sojourn time in the duodenum is very short, on the order of minutes[4]. When the steady-state luminal calcium is low and transcellular transport is near its maximum, a significant proportion of luminal calcium in the duodenum is absorbed and only relatively small quantities of calcium remain available for paracellular absorption in jejunum and ileum, even though sojourn times there are of the order of hours. But when the calcium supply is abundant, most of the calcium in the lumen of the duodenum will escape absorption there and be available for paracellular transport in jejunum and ileum. Moreover, the concentration gradient between luminal and body fluid calcium will be much greater on a high than on a low calcium intake. It is thus evident that calcium entry into the body is essentially unrestricted.

Calcium is lost from the body by two principal routes, via urine and the endogenous calcium that enters the intestine. There, it mixes and undergoes reabsorption with food calcium. Unabsorbed food and endogenous calcium is excreted in the feces. The net loss of endogenous calcium in adults approximately equals the loss in urine[1]. Urinary calcium varies with calcium intake[1]. The kidneys, therefore, do play a regulatory role, but it is not a major one. It has been shown[15] that if the kidneys were the only organs disposing of calcium, the disposal half time required is much greater than the actual half time in which the body disposes of a calcium load. For example, in a rat, the observed half time for disposing of a calcium load is approximately 14 min[15], whereas it would take some 30 times longer if the kidneys were to act alone in disposing of the load. In other words, plasma calcium regulation resides only minimally in the kidney or in the disposal of endogenous calcium in the stool, inasmuch as the amount in and turnover of the latter is about the same as in urine.

This, then, leaves bone as the major regulator of extracellular calcium. This inference is supported by the calculation that the mammalian skeleton can dispose of half a calcium load in 27 circulations; this amounts to some 10 min in rats and some 25 min in humans[15].

How does bone regulate plasma calcium? Bone formation and resorption are, as well known, cellular processes. While these processes are initiated very rapidly, their end products—formation or resorption of bone elements—take substantially longer than the rate at which either positive or negative calcium loads are cleared from the circulation[15]. Instead, Wilfred Stein and I have proposed that there exists a rapid and dynamic equilibrium between bone calcium binding sites and plasma calcium This equilibrium can be described by an apparent half-saturation concentration, $K_m$, with $K_m$ equal to the plasma calcium concentration.

Bone contains a variety of calcium salts, including brushite, octacalcium phosphate, amorphous calcium phosphate, whitlockite, with hydroxyapatite, the least soluble of these salts, being the most mature form of skeletal calcium[16]. It is not hard to imagine that the solubility of these salts parallels their molar Ca/P ratio and that bone therefore contains a multiplicity of calcium binding sites, with brushite having the lowest and hydroxyapatite the highest affinity for calcium. Low-affinity sites of bone mineral are therefore associated with and located in the vicinity of osteoblasts, whereas high-affinity sites are found in the mature regions of bone, to the vicinity of which active osteoclasts are attracted in response to osteoblast signals.

To regulate plasma calcium requires that the $K_m$ can change rapidly. Such a rapid change can be brought about if there is a change in the proportion of high- and low-affinity binding sites. An increase in the number of available high-affinity binding sites would then lower the $K_m$ and plasma calcium. An increase in the number of low-affinity binding sites would increase $K_m$ and plasma calcium.

We propose that these rapid changes are brought about by shape changes in bone cells[15,17]. Thus the earliest response of osteoblasts to an increase in parathyroid hormone is contraction or balling up of the osteoblasts, causing more bone surface to become available[18]. At the same time, the metabolic activity of the osteoblasts is reduced or inhibited. In response, osteoclasts may at the same time spread out on signals from the osteoblasts, so that more high-affinity sites are also blocked. The result is a rise in plasma calcium. Similarly, release or injection of calcitonin will cause osteoclasts, equipped with calcitonin receptors, to contract[18], exposing more high-affinity calcium binding sites, with plasma calcium therefore dropping. Changes in the appropriate signals will cause osteoblasts to expand and cover more low-affinity binding sites. This will contribute to lowering plasma calcium. Again, cell contraction leads to a slow-down or transient inhibition of some metabolic processes, with bone formation or resorption ultimately diminished (Fig. 1).

Even if not all details of the events described above are ultimately confirmed experimentally, it is, I believe, indisputable that the major regulation of extracellular calcium is exerted via the calcium binding sites of the bone mineral and the action thereon by the cells of bone.

In the cell, there are many fixed calcium-binding sites: in mitochondria, in rough and smooth endoplasmic reticulum, in other stationary organelles. These may be considered the functional equivalent of the skeleton. Their cellular calcium-binding capacity is fairly large, as tripling the extracellular calcium *in vitro* from 1 to 3 mM only doubled cellular calcium uptake, with the cells remaining viable[19]. Many studies have shown that these fixed binding sites not only take up

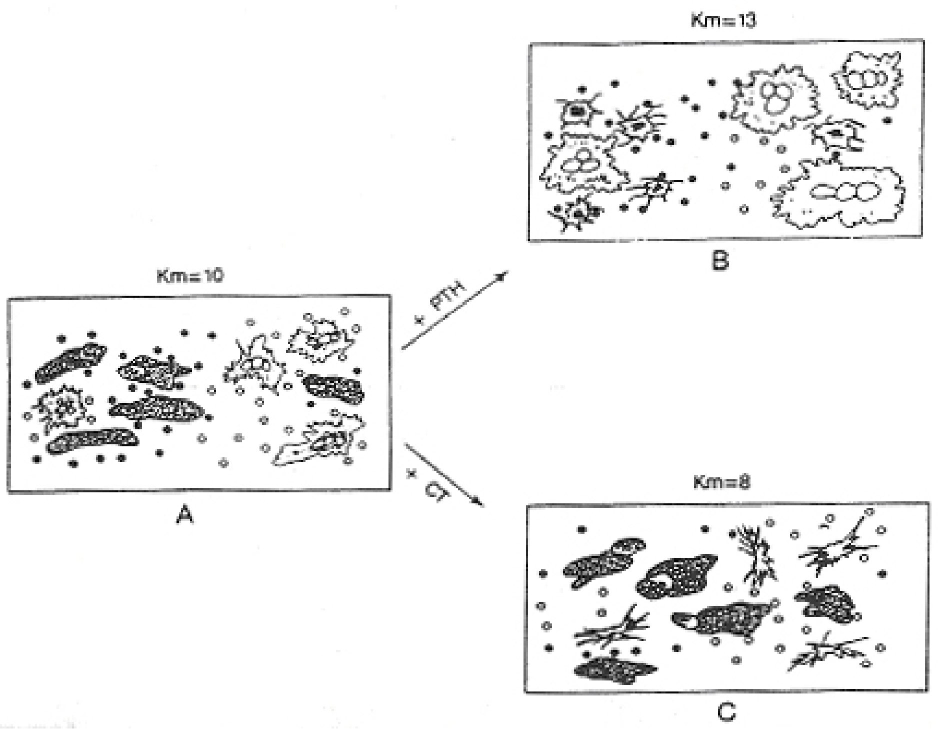

**FIGURE 1.** Diagram representing the effect of the acute administration of parathyroid hormone (PTH) or calcitonin (CT) on the spatial relationships of osteoblasts and osteoclasts on a bone surface. The open and closed circles represent, respectively, the $K_m$ values of the high-affinity bone calcium-binding sites. (A) Represents a normocalcemic situation, with an equal number of high- and low-affinity sites. (B) Represents the result of PTH administration, where the shrinkage of osteoblasts has exposed low-affinity sites and the associated expansion of osteoclasts has blocked high-affinity sites, leading to an average $K_m$ of 13 and hypercalcemia. (C) Represents the result of CT administration, where the shrinkage of osteoclasts has exposed high-affinity sites and the consequent expansion of osteoblasts has blocked low-affinity sites, leading to an average of $K_m$ of 8 and hypocalcemia. Note: High-affinity sites are considered to have an apparent $K_m$ of 5 mg Ca/dl, with low-affinity sites an apparent $K_m$ of 15 mg Ca/dl. For the sake of convenience, the $K_m$'s refer to total plasma calcium, rather than the theoretically correct ionic calcium concentration, approximately half of the total. Bone mineral with a high Ca:P ratio is considered to have a relatively higher affinity for calcium binding than bone mineral with a low Ca:P ratio (see text and Table 1). Reproduced with permission of Bronner and Stein[17].

calcium, but also release it. Often intracellular calcium signals involve calcium released from binding sites, which then become replenished by extracellular calcium[20]. But, in contrast with the body as a whole, which exists in a relatively low calcium environment, the cells of the body are forced to maintain a very low intracellular calcium concentration, while at the same time surrounded by the far higher calcium concentration that characterizes the body fluids. If cells did not regulate calcium entry severely and strictly, they would be flooded with calcium and their intracellular calcium concentration would rise from the nanomolar to the millimolar level.

Current research has identified two mechanisms of intestinal cells that monitor external calcium concentration, a calcium-sensing receptor (CaR, [21,22,23]) and an apical entry structure, termed ECaC[11,12] and CaT1[13]. The calcium-sensing receptor provides a signal that modulates secretomotor functions and/or epithelial growth and differentiation. The entry structure (ECaC and CaT1) appears to have two mechanisms for calcium entry, a transporter mechanism that responds to and saturates at a low luminal calcium level and a channel mechanism that is regulated by intracellular calcium, with calcium flux proportional to luminal calcium. Analysis of transcellular calcium flux in the duodenum[14] has shown that in the absence of vitamin D calcium entry is reduced to about 7% of what it is in the presence of the vitamin. This analysis finds support in a recent publication[5].

Effectively then the nontransporting cell resists calcium flooding by severe entry restriction. The transporting cell, on the other hand, when under the hormone-like action of vitamin D, increases calcium entry via its channel component, with calbindin $D_{9k}$ buffering and ferrying the incoming calcium. The calcium extrusion capacity of duodenal cells appears adequate to handle calcium flux at its maximum[2,3]. However, as mentioned above, the number of CaATPases may be increased as a result of the action of vitamin D[7].

## CONCLUSION

Two principal mechanisms have been utilized by the whole body and the cell to regulate calcium concentration. The body as a whole can restrict calcium entry by downregulating active transcellular calcium transport in the duodenum. However, if calcium intake is abundant, the overall effect of this downregulation is modest, inasmuch as paracellular absorption proceeds in the small intestine. The major regulatory mechanism of the body is the skeleton, in which calcium is deposited and from which calcium is removed by isoionic exchange, a process modulated by bone cells that in turn are subject to hormonal action and regulation. The individual cell, however, bathed in a calcium medium that is $10^4$ times higher than the average intracellular calcium concentration, must severely restrict calcium entry. Once calcium enters the cell, fixed intracellular organelles take up and release calcium in analogy with how the skeleton functions. Intestinal cells that transport calcium are equipped on the apical pole with an entry structure that functions as both transporter and channel. This structure enables the cell to upregulate calcium entry, with intracellular transport mediated by the mobile, vitamin D–dependent buffer, calbindin $D_{9K}$.
